# The Serum Diagnosis of Hog-cholera1Reprinted from the New York Medical Journal of February 20, 1897.

**Published:** 1897-05

**Authors:** Charles F. Dawson

**Affiliations:** Bureau of Animal Industry, Washington, D. C.


### SELECTIONS.

### THE SERUM DIAGNOSIS OF HOG-CHOLERA.[1]

By Charles F. Dawson, M.D., D.V.S.,
BUREAU OF ANIMAL INDUSTRY, WASHINGTON, D. C.

In July, 1896, Widal[2] described a method which he had recently discovered for diagnosticating with ease and certainty the existence of typhoid fever.

The importance of such a discovery is apparent, since by this means physicians can ascertain the existence of this disease with comparative ease. The method is as follows:

A bouillon culture of the typhoid bacillus is examined microscopically to determine the motility and isolation of the individual bacteria. A few drops of the culture are placed in a watch-glass

[1] Reprinted from the New York Medical Journal of February 20, 1897.

[2] La Presse Médicale, July 29, 1896.

and with this is mixed a drop of blood drawn from the finger-tip of the patient. Hanging-drop preparations are then made from the mixture of culture and blood. A most interesting series of phenomena presents itself. The bacilli are not isolated and moving as they do in ordinary cultures, but lose their motility and become agglutinated and joined together in masses which are separated by wide spaces. The clear spaces are dotted with less motile bacilli, and these can be seen approaching the masses and finally adhering to them. Widal observed these phenomena in preparations made from patients in different stages of the disease. They did not appear in preparations made from the blood of persons in health, and he argues therefrom the reliability of the test. Nor did the phenomena appear in preparations made from patients suffering with other diseases—such as nephritis, tuberculosis, pneumonia, icterus, and rheumatism; but the bacilli remained motile, and exhibited no tendency to become non-motile and massed together in clumps.

These very interesting results determined the writer to apply the test in hog-cholera, a disease resembling typhoid fever in many respects. In these observations, which are preliminary to a more extended investigation of the subject, hog-cholera was induced in a rabbit by the subcutaneous injection of a bouillon culture.

On the fifth day after inoculation a small piece of the ear of the rabbit was excised and clean cover-glasses were smeared with the small amount of blood which oozed from the wound. No attempt was made to prevent the drying of the preparations, and the result obtained would indicate that evaporation of the liquid portion of the blood does not prevent the appearance of the characteristic phenomena. When the preparations were perfectly dry a drop of bouillon culture of the hog-cholera bacillus was placed upon the stratum of dried blood on the cover-glass, which was then inverted over a hollow-ground slide and examined carefully with a Zeiss two-millimetre apochromatic immersion lens combined with a No. 4 ocular. By the time the slide was prepared and focussed the bacilli, which had been previously observed to be motile in a control preparation, became motionless and agglutinated in clumps, exactly as was described by Widal in reference to the typhoid bacilli. A control experiment was made with the blood from a normal rabbit, with the result that the hog-cholera bacilli did not exhibit the slightest tendency to be affected in any way. Similar experiments were made to determine the effect of hog-cholera blood-serum upon the typhoid fever bacillus and the *Bacillus coli com-*

*munis*. The results were negative. The absence of any effect of the hog-cholera serum upon the typhoid bacillus and also upon the *Bacillus coli communis* is of interest on account of the resemblance of these three organisms in other ways.

Owing to the obscurity of the symptoms usually presented in hog-cholera, it would seem that a method which would render a correct diagnosis would be of considerable value, it being by no means an easy task to diagnosticate hog-cholera from the physical symptoms alone. As it is possible that a cure or preventive of hog-cholera based upon antitoxic-serum therapy will be discovered, it would be of great advantage to have a method for detecting the existence of the disease in its incipiency.

While the experiments upon rabbits are not offered as positive evidence that the same results could be obtained from affected hogs, it is reasonable to assume that a similar result is within the range of probability.

